# Notes and News

**Published:** 1887-08-06

**Authors:** 


### NOTES AND NEWS.

COLLECTED AND COMMUNICATED.

The Jubilee Fund for the Aberdeen Royal Infirmary now amounts to £24,109 16*s*. 9*d*.

It is proposed to erect an infectious hospital at Stourbridge, for the use of the inhabitants of that town and the adjoining districts.

The railway servants at Carlisle have held their annual entertainment on behalf of the Cumberland Infirmary. The entertainment was excellent, and included some of the most popular songs of the day, as well as selections by Mr. Williams, a celebrated local player on the concertina.

A meeting of the committee of the Cotton Districts Convalescent Fund was held in the Town Hall, Manchester, under the presidency of the Earl of Derby, K.G. An application had been made on behalf of the Burnley Victoria Hospital to be affiliated with the fund, and Mr. Joshua Rawlinson, J.P., the honorary secretary to the hospital, and Mr. James Kay, J.P., a member of the Board of Management, attended as a deputation in support of the application. Lord Derby said that the committee of the fund were glad to accede to the application made on the part of the hospital. It is expected and believed that the Victoria Hospital will derive great advantages from this connection, as convalescent patients can now be sent to both Southport and Buxton.

During the past year the Royal Isle of Wight Infirmary has been enabled by special contributions to keep open twelve extra beds. The committee, through their chairman, A. H. Tattnall, Esq., and the Venerable Archdeacon Haigh, make a further appeal for additional contributions, with the object of maintaining twelve extra beds in the present year. Several liberal donations have already been promised.

It appears that the Beau Site Convalescent Home at Hastings has been maligned. Mr. H. J. Lias, the chairman of the house committee, writes to a local paper, stating that "the Home has a good and sufficient balance at the bank," and not only so, but "is well and generously supported." Notwithstanding this necessary contradiction of an unauthorised report, Mr. Lias states that the committee appeal to the public for a regular and permanent annual income upon which they can rely.

The nursing staff of the Leicester Infirmary have held a bazaar for the purpose of raising funds to provide a library for their use, and to improve the organ in the chapel. We are glad to know that the public have taken up the question with much enthusiasm and sympathetic feeling. The nurses at the Leicester Infirmary will, we hope, in their new quarters enjoy a degree of health and comfort which was scarcely possible formerly. The desire for intellectual improvement, shown by this effort to obtain a library, deserves the warm sympathy of all friends of hospitals.

On Wednesday the new building of the Hull and Sculcoats Dispensary was opened. At the ceremony there were present the Right Worshipful the Mayor, Alderman Leak, the Worshipful the Sheriff, Dr. Sherburn, Alderman Willows, ex-mayor and chairman of the committee, Mr. W. R. King, J.P., Messrs. Haller, Bannister, Botteril, and Bilston, architects, Mr. Blackburn, contractor, Mr. Legg Roe, house-surgeon, and many others. At the same time a bust of the late Mr. Thomas Dibb, chairman of the institution, was unveiled.

"Probably the last echo of our recent Jubilee festivities," says the *Birkenhead News*, "died away in the wards of the Wirral Children's Hospital on Wednesday evening, when the celebration was held that should have taken place on June 21st." The ceremony was of rather a romantic character, the idea being to reproduce the procession of the Queen's Coronation, the dresses being copied from those shown in a celebrated painting of that event. The procession was a highly picturesque sight. First came the trumpeters, walking backwards: then the lady who personated her Majesty, clad in a dress of white net lace with ruby velvet train, trimmed with gold and decorated with all the insignia of royalty. The train-bearer was a pretty little lad in peach-coloured satin coat, white satin breeches, silk hose, shoes trimmed with bows to match cape, white felt-hat with long ostrich feather, and sword, all complete. The remainder of the procession was strictly in keeping and was a great success. Every child in the hospital received a present of a commemoration medal, the gift of Dr. Braidwood.

The following vacancies are announced this week, the amount of the salary being placed in each case after the name of the institution:—

**Resident Medical Officers.**

Durness, Sutherlandshire. Parish Medical Officer.—£150.
Leeds Infirmary One, qualified.—£100.
Sunderland Infirmary. House Physician. Doubly-qualified.—£80.
Tottenham Hospital. Assistant House Surgeon. Doubly-qualified.—No salary. Address, Dr. Laseron, Tottenham Green.
Ventnor Consumption Hospital. Assistant. Fully qualified.

**Non-resident Medical Officer.**

West End Hospital for Paralysis, etc., 73, Welbeck Street. Physician, to attend two evenings a week.

**Matrons, etc.**

Glasgow Western Infirmary. Matron's Assistant.
Manchester, Monsall Fever Hospital. Lady Superintendent.—£80. Apply, The Secretary, Manchester Infirmary.

**Secretary, etc.**

Norfolk and Norwich Hospital.

**Trained Nurses.**

Bideford Union. One.—£20.
Chesham Cottage Hospital.—£30.
City of London Hospital for Diseases of the Chest. Day Nurse.—£22.
Great Yarmouth Hospital. Night Nurse.—£18.
Kingston Home, Hull. Several
Miller Hospital, Greenwich.—£22.
Seamen's Hospital (late Dreadnought). One.—£20.
West London Hospital. Assistant.—£24.

**Probationers.**

Cork Hospital for Diseases of Women and Children. Two.
Miller Hospital, Greenwich. One.

---

The second annual church-parade in aid of the funds of the Richmond Hospital, organised by the Castlegate Lodge of the Total Abstinent Sons of the Phœnix, took place on Sunday afternoon. The report goes on to state that the church was not full, for some of the processionists preferred to remain outside—a curious termination to a church-parade. Notwithstanding this peculiar attitude of the processionists, the Rev. E. Talbot preached an excellent sermon, and the amount collected in the church and in the boxes outside was £14 16*s.* 1¾*d.* We are glad that those outside did not run away from the collection as they did from the service.

---

Miss Ross, of Granville College, entertained the inmates of the Whitgift Hospital at tea on Saturday. The young ladies of the school were assiduous in their attentions, as well as also many visitors who were present. After tea there was a concert of vocal and instrumental music, with recitations, which gave infinite pleasure to the audience. There was also a well-got-up little theatrical performance by the young ladies, of "Alfred the Great and the Cakes," which elicited roars of laughter. It would be hard to decide which seemed to enjoy the entertainment most, the hostesses or their guests.

---

On Friday the Lord Mayor of London laid the foundation stone of a new hospital at Blackpool, Lancashire, to be called the Victoria Hospital. There were present a large number of provincial mayors and other gentlemen. A procession was organised from the Imperial Hotel to the site of the new building, and Volunteer and other bands were in attendance. The weather was magnificent, and the whole route of the procession lined with thousands of spectators. The ceremony was of a most imposing character.

There is a growing feeling among officers of the Naval Medical Service that their interests are not studied as they should be, and that they are at a distinct disadvantage as compared with their brethren in the army. That this should be so is not a little strange, seeing that the two services have to appeal to exactly the same class for candidates; and as life at sea as a non-executive officer has many drawbacks, it is more than probable that the navy now runs the risk of obtaining as surgeons some of those who failed to pass in the army. Red-tape and officialism are constantly bungling, and that they should bungle on this subject, of which they know little, and probably care less, is not surprising, though much to be regretted.

---

At Swindon a misunderstanding arose at the time of the laying of the foundation stone of the new hospital, which caused the Nonconformist ministers of that town to absent themselves in a body from the ceremony. In most places it is found possible to sink religious differences as the hospital-doors are approached. Whoever has been the cause of this unfortunate interruption of good feeling is highly reprehensible. We sincerely hope that after the first feelings of anger have subsided better counsels may prevail, and that the religious bodies may manifest by their example, as well as by their professions, that charity which is the chiefest of all the Christian virtues.

---

At Wokingham, for several years past, the services of one of the trained nurses of the Royal Berks Hospital have been secured for the benefit of the working-classes and those immediately above them at a considerably reduced charge. A local financial committee provides the lodging for the nurse and collects subscriptions, but the disposal of her services rests entirely with the medical men of the town. The plan adopted is that the nurse calls on them each morning and receives their directions. Special attention is given to anxious cases, but her time is not devoted to any particular patient, nor does she sit up at night except at the request of the doctor. The Hospital Board have satisfied themselves that this arrangement is completely successful, and with the hope of inducing other parishes, towns, and districts to adopt similar measures, they have reduced the payment to £30 a year.

---

The *Bombay Gazette* says: "Mr. Tharia Topun made an offer to the British Government on the day of the celebration in Bombay of Her Majesty's Jubilee in February last, to build and endow a hospital at Zanzibar. The estimated cost of building the hospital will be about 80,000 rupees, and Mr. Tharia Topun has offered a further sum of 150,000 rupees for maintaining the institution on a permanent basis, which amount the British Government will hold in trust for the donor. The institution will be named the Tharia Topun Hospital. The offer has been accepted by Lord Salisbury, as Foreign Minister, on behalf of Her Majesty's Government. Mr. Tharia Topun has traded with the eastern coast of Africa for the last fifty years. Mrs. Tharia Topun will, soon after the monsoon, start for Zanzibar to lay the foundation stone of the hospital, and superintend the building herself."

The Vicar of Croydon and Miss Braithwaite on Thursday afternoon gave their annual garden party to the inmates of the Whitgift Hospital. A splendid tea was served on the lawn, during which a local string-band was in attendance, and discoursed sweet music. Amongst those present were the Mayor and Mayoress of Croydon, and many visitors and friends of the host and hostess.

---

On Wednesday Earl Sondes opened a bazaar in aid of the Kent and Canterbury Hospital. A large number of the ladies of the district presided at the stalls, and there was a great variety of entertainments. In addition to other instrumental music, the band of the "Buffs" from Dover was in attendance. The railway companies ran special trains, and no efforts were spared to bring a large number of visitors to the town.

---

The annual meeting of the Dockyard Shipwrights' Hospital Fund was held in the hall of the Chatham Workmen's Club on Wednesday evening. The chair was occupied by Mr. Clum ; and Mr. Haygie, secretary, and Mr. R. England, treasurer, were also present. The chairman said they must be all agreed that Mr. Haygie had presented them with a favourable report. He thought that the secretary had the entire confidence of the whole of the shipwrights in the dockyard. After the passing of the accounts Mr. Haygie was re-elected secretary, and Mr. England treasurer of the fund.

---

On Saturday, the 23rd ult., the guardians of the Lancaster Union had under consideration the question of providing hospital accommodation for the rural districts of the Union. There seems to be much difference of opinion on the subject, not only among the guardians themselves, but outside, and they have, therefore, wisely determined to refer the matter for consideration to a committee specially appointed for the purpose.

---

Referring to the late Dr. A. H. Bartlet, the Rev. G. Fraser Wheland, curate of St. Peter's, Ipswich said : "The topic of this day reminds us of the death of one who had been for so many years a resident in this parish, and who was a governor of the Ipswich and East Suffolk Hospital, as well as a munificent subscriber to its funds, for I see from the last report that he gave two donations of over £110 to relieve the wants of the sick in connection with the hospital. Of course you are all aware that I refer to the late lamented Dr. Bartlet. To many of you he was no doubt only known as an eminent man who stood high in that noble, that humane profession of medicine which he had chosen. He was also a liberal supporter of other charities in the town, and I may say I think it due to his memory that I have heard from one who knew him well that few had the slightest idea of his many deeds of kindness and love among the people, for those deeds were done privately, and without ostentation. I am only speaking the sincere feelings of my heart when I say it was a true pleasure to me to visit him ; he always received me most kindly, and listened reverently to the teaching of the Sacred Book, but now, full of years, having attained to a ripe age, he rests, we trust, with Christ."

At Stockton-on-Tees, as at Huddersfield and other places, there is an annual demonstration of Friendly and Trade Societies. The third demonstration was recently held, when a procession was formed and marched to the theatre. The Mayor, Mr. Joseph Dodds, M.P., Mr. W. D. Stephens of Newcastle, and Mr. William Snow, each delivered a short address. Collections were taken all along the road and in the theatre, and more than five-and-twenty pounds was available for allocation after the proceedings terminated.

---

Even at a small town like Beverley a similar annual demonstration to the above produced no less a sum than £40.

---

There is no Convalescent Home in connection with the Fever Hospital at Edinburgh, which is somewhat surprising, considering the position that Edinburgh holds in the medical world, and not very creditable. A sub-committee of the Public Health Committee of the Town Council have had under discussion the proposal to establish such a Home. Three different methods have been under consideration. According to the first, patients on leaving the hospital would be boarded out in the country at the public expense ; or, secondly, it is proposed to buy or build a house of sufficient accommodation in the suburbs ; or, thirdly, a small permanent Home, to be supplemented by the boarding-out system, is suggested. The sub-committee have resolved to make a recommendation to the Public Health Committee in terms of the last-mentioned proposal.

---

According to Col. King-Harman, the salaries at the Dundrum Asylum have increased at the following rates since 1850. The original salary of the resident physician and governor was £240 a year : it is now £450. The visiting physician had a salary of £150 a year, which has been increased to £175. In 1875 the Protestant chaplain was paid at the rate of £40 a year : since that date he has received £50 a year. The Roman Catholic chaplain in 1875 had £60 a year, and now has £80. The head male attendant, whose original salary was £24, has now £60. The subordinate staff, composed of attendants and ordinary domestics, who received at the earlier date £12 on an average, are now paid £18 a year. The total of the increased rate of remuneration appears to be about £450.

---

At Totnes a fête and bazaar were held on the island, lent for the occasion by the Duke of Somerset, on the 20th and 21st ult., in aid of the funds of the Cottage Hospital. The stalls were presided over by Mesdames Champernowne, T. H. Edmonds, Everett, A. Pike, Currie, Tollit, T. Maye, L. Hains, J. E. Lloyd, and Fraser ; the Misses Middleton, Miss Edith Champernowne, and Miss Bond. The amusements included theatrical performances, Punch and Judy, Aunt Sally, magic wells, fish-ponds, a flower-show, and other attractions. The Totnes Volunteer Band was present, and in the evening there was a grand promenade concert. The fête and bazaar were under the patronage of the Duke of Somerset, the Mayor, Mr. H. Symons, Revs. B. Champernowne and A. J. Everett, Major Rowland, and other gentlemen.

